# Structural and Biomechanical Corneal Differences between Type 2 Diabetic and Nondiabetic Patients

**DOI:** 10.1155/2019/3764878

**Published:** 2019-03-18

**Authors:** João N. Beato, João Esteves-Leandro, David Reis, Manuel Falcão, Vítor Rosas, Ângela Carneiro, Fernando Falcão Reis

**Affiliations:** ^1^Department of Ophthalmology, São João Hospital, Porto, Portugal; ^2^Department of Surgery and Physiology, Faculty of Medicine, University of Porto, Porto, Portugal; ^3^Faculty of Medicine, University of Porto, Porto, Portugal

## Abstract

**Purpose:**

To analyze and compare corneal structural and biomechanical properties, characterized by corneal hysteresis (CH) and resistance factor (CRF), between patients with and without type 2 diabetes mellitus (DM), and determine the main ocular variables that influence them.

**Methods:**

Sixty diabetic and 48 age- and sex-matched non-DM patients were enrolled in this cross-sectional study. The DM group was analyzed according to DM duration (<or ≥ 10 years), HbA1c levels (<or ≥ 7%), and presence of retinopathy. CH and CRF were evaluated using the Ocular Response Analyzer® (ORA). Central corneal thickness (CCT) was determined by Scheimpflug tomography (Pentacam® HR). Intraocular pressure was obtained with ORA (IOPcc) and Goldmann applanation tonometry (IOP-GAT). Univariate and multivariate linear regression analyses were performed to evaluate the relationship between demographical, clinical, and ocular variables with the biomechanical properties.

**Results:**

There were no statistically significant differences in the CH and the CRF between DM and non-DM groups (*p*=0.637 and *p*=0.439, respectively). Also, there was no statistical difference between groups for the CCT, IOPcc, or IOP-GAT. Multivariate linear regression analysis showed that CH was positively associated with CCT (*p* < 0.001) and negatively associated with IOPcc (*p* < 0.001), while CRF was positively associated with CCT (*p* < 0.001) and IOPcc (*p*=0.014).

**Conclusion:**

The CCT and IOPcc were found to be the main parameters that affect corneal biomechanical properties both in diabetics and controls. In this study, there was no significant effect of DM type 2 on corneal biomechanics.

## 1. Introduction

In the last decade, there has been a growing interest in the study of corneal biomechanics suggesting that the cornea acts as a viscoelastic structure that might be influenced by ocular and systemic conditions [1].

In clinical practice, corneal biomechanical properties can be easily and accurately [2, 3] estimated using the Ocular Response Analyzer® (ORA). It evaluates corneal deformation response though a calibrated air puff and calculates corneal hysteresis (CH) and resistance factor (CRF) [4]. CH predominantly reflects the viscoelastic response of the cornea to an applied force defined by a specific air-pressure curve [5], whereas the CRF provides information on overall resistance of the cornea to deformation. It is important to note that CH and CRF are not directly related, and alterations in tissue structure can lead to independent changes in both parameters [6]. The ORA also provides a biomechanically adjusted estimate of intraocular pressure (IOPcc) that is less affected by corneal thickness or curvature [7].

Recent evidence has shown that the central corneal thickness (CCT) only accounts for a small fraction of the variance in IOP when compared to the biomechanical properties of the cornea. In fact, CH was found to be more strongly associated with glaucoma presence, risk of progression, and effectiveness of glaucoma treatments than CCT [8].

The relationship between corneal morphological changes and elevated plasma glucose concentrations in diabetes mellitus (DM) has been extensively reviewed. Most importantly, the presence of hyperglycemic states can cause a nonenzymatic glycosylation of collagen, proteoglycans, and glycosaminoglycans (Maillard reaction) that results in increased corneal stiffening [9].

Clinical investigations have shown that adult diabetic subjects may have altered corneal biomechanics [10–18]; however, this relationship is far from being clarified. For example, CH was reported to be greater in DM patients compared to non-DM subjects [11–15], while others reported no differences or significantly inferior values [16, 17, 19]. One reason for this is that different studies had different definitions of DM (by interview or glycated hemoglobin A1c (HbA1c) levels), selection criteria (DM type and severity), and designs (control of confounding factors such as IOP and CCT), resulting in contradictory results. Also, no studies have addressed eventual corneal biomechanical associations to different stages of diabetic retinopathy (DR).

The purpose of this study is to evaluate the differences in corneal structural and biomechanical properties between patients with and without type 2 DM. In addition, it aims to determine the main ocular variables that influence the corneal biomechanics.

## 2. Methods

### 2.1. Subjects

A cross-sectional observational study was performed. Type 2 diabetic patients with different stages of DR and controls, aged 50 or older, were prospectively recruited from the Cataract and Refractive Surgery Unit of the Ophthalmology Department of Centro Hospitalar São João between September 2015 and March 2016. Medical records of all patients scheduled for monocular phacoemulsification cataract surgery were reviewed, and eligible patients were invited to participate. Informed consent was obtained from each participant before inclusion in the study. The study protocol adhered to the tenets of the Declaration of Helsinki and received Institutional Review Board approval.

The diagnosis of type 2 DM was based on the medical history, HbA1c levels ≥6.5%, and/or current use of antidiabetic medication [20]. Nondiabetic age- and sex-matched patients were used as controls. All study participants were Caucasian. The same exclusion criteria were used for both groups, and they included prior eye surgery (except for anti-VEGF agents or triamcinolone intravitreal injections < 120 days or laser photocoagulation < 90 days before surgery in the study eye of diabetics), any corneal, retinal, or optic nerve disease except DR (e.g., glaucoma, age-related macular degeneration, vascular occlusions, uveitis, and other chorioretinal diseases), mature cataracts (nuclear opacity grade greater than 3, from 1 (mild) to 4 (white/brown) severity grading system), Goldmann applanation tonometry (IOP-GAT) > 25 mmHg, pseudoexfoliation syndrome, current treatment with glucocorticoids, and ORA waveform score (WS) ≤ 3.5 [21]. Diabetic patients were excluded if they had uncontrolled complications of proliferative DR (e.g., current iris neovascularization, vitreous hemorrhage, or tractional retinal detachment). All patients with a serious illness or syndrome and any physical or mental problem that could hinder the examinations required for the study were also not included.

### 2.2. Study Protocol

All subjects underwent a standard examination which included a general anamnesis to obtain demographical and medical history (ocular and systemic). Before measurements, each participant was subjected to a complete ophthalmic evaluation performed in a standardized fashion by the same ophthalmologist. The grade of DR was assessed in all diabetic patients using 7 standard ETDRS fundus photographs [22].

The examinations were sequentially performed with the IOLMaster® 500 (software version 7.7) and then with the Pentacam® HR Scheimpflug tomographer (Pentacam HR version 6.08r19 with the software version 1.20r87). Measurements were repeated as necessary until high-quality images were obtained. Only good-quality examinations were accepted, defined as scans that passed the software's quality check.

Corneal biomechanical properties were assessed using the ORA (software version 1.1). After checking for a good alignment of the eye and the probe, a series of four good-quality measurements was performed on both eyes of each subject. For each eye, the measurement with the higher waveform score was used for the analysis, as recommended by the manufacturer.

All measurements were performed by an experienced operator (JB) in a darkened room between 1 and 7 pm, without cyclopegia, and the patients were told to blink immediately before each examination.

Following instillation of topical corneal anesthesia (oxybuprocaine hydrochloride 0.5% with fluorescein sodium 0.25%), the IOP was measured twice by a masked investigator (JEL) using the Goldmann applanation tonometer (GAT).

At the end of the visit, an experienced nurse evaluated all the individuals to record vital signs and collect blood samples, by venous puncture, for serum HbA1c analysis. These allowed the authors to evaluate the glycemic status of DM patients and disclose undetected diabetes in the non-DM group.

### 2.3. Devices

#### 2.3.1. IOLMaster® 500 (Carl Zeiss Meditec, Jena, Germany)

The IOLMaster is a partial coherence interferometer used for optical biometry. It measures the AL (mean of five measurements) through an infrared light (780 nm) and has been shown to have high intraobserver and interobserver reproducibility [23].

#### 2.3.2. Pentacam® HR (Oculus, Wetzlar, Germany)

The Pentacam uses a single 180-degree rotating Scheimpflug camera and a monochromatic slit-light source (blue LED at 475 nm) combined with a static camera (for the correction of any eye movement) to generate a three-dimensional high-resolution (HR) image of the anterior segment. Anterior keratometry and apex paquimetry (CCT) have been shown to have excellent repeatability and reproducibility [24].

#### 2.3.3. Ocular Response Analyzer® (Reichert Ophthalmic Instruments, New York, USA)

The ORA is a noncontact tonometer that uses a calibrated air puff and infrared electro-optical system to measure the required force to flatten the cornea as the air pressure rises (force-in applanation, P1) and the force at which the cornea becomes flat again as the air pressure falls (force-out applanation, P2) [4]. It determines four basic parameters based on the 2 pressure measurements at applanation. The difference between P1 and P2 is called CH and represents the viscoelastic properties of the cornea. The average of P1 and P2 is called Goldmann-correlated IOP (IOPg). Through empirical investigation, 2 other parameters, calculated as a linear function of both applanation pressures, were defined: corneal resistance factor (CRF), which is supposed to be more correlated with CCT, and corneal-compensated IOP (IOPcc), which was designed to be similar before and after refractive surgery [5].

### 2.4. Sample Size Calculation

For a type I error of 0.05 and type II error of 0.20 (80% power), considering a mean difference of CH ≥ 1 mmHg to be significant and assuming the SD for the non-DM group of 1.7 mmHg [10, 16, 17], the minimal required sample size would be 46 subjects in each group. We included additional patients in the DM group in order to perform subgroup analysis.

### 2.5. Data and Statistical Analysis

Diabetic subjects were classified into subgroups according to DM duration (<10 and ≥10 years), HbA1c levels (<7.0 and ≥7.0%), and DR (absence or presence of DR). According to patient self-reports, smoking status was evaluated (never smokers and active/former smokers groups). Body mass index (BMI, in kg/m^2^) was calculated as weight/height^2^ using measured weight and height.

Statistical analysis was performed using the SPSS® statistical software (version 21.0 for Mac OS; SPSS Inc., Chicago, IL, USA). In the present study, only the fellow nonscheduled eye of each patient undergoing monocular cataract surgery was used for statistical analyses. The Kolmogorov–Smirnov test and normal probability plots were used to confirm the normal distribution of the data. Parametric or nonparametric tests were used for continuous variables comparison between the DM and non-DM groups, according to the normality of data. Chi^2^ or Fisher's exact tests were performed for categorical variables comparison. Univariate and multivariate linear regression analyses, using generalized linear models, were performed to identify the potential demographical/clinical (age, gender, body mass index (BMI), DM duration, HbA1c levels, and smoke history) and ocular variables (AL, Km, CCT, IOPcc, and IOP-GAT) associated with CH and CRF. Statistical significance for all the analyses was set at a *p* value less than 0.05.

STROBE guidelines were followed for manuscript elaboration [25].

## 3. Results

Sixty diabetic patients and 48 nondiabetic controls were enrolled in the study. Demographic and clinical characteristics of the study population did not show any significant differences between groups, except for the levels of HbA1c (Table 1).

In the DM group, duration of DM was significantly associated with HbA1c levels (*p* = 0.004, chi^2^ test) and severity of DR (*p*=0.014, Fisher's exact test), as well as severity of DR and HbA1c levels (*p*=0.028, Fisher's exact test).

### 3.1. Comparison of Ocular Parameters between DM and Non-DM Groups

There were no significant differences between groups for any of the studied variables IOP-GAT, IOPcc, CH, and CRF (Table 2).

### 3.2. Subgroup Analysis of Corneal Biomechanics in the DM Group

#### 3.2.1. Duration of Diabetes

There were no statistically significant differences in the CH and the CRF between the DM group ≥10 years and <10 years (*p*=0.233 and *p*=0.189, respectively) (Table 3).

#### 3.2.2. HbA1c Levels

There were no statistically significant differences in the CH and the CRF between the DM group with HbA1c ≥ 7% and HbA1c < 7% (*p*=0.507 and *p*=0.228, respectively) (Table 3).

#### 3.2.3. DR Stage

There were no statistically significant differences in the CH and the CRF between DM groups with and without retinopathy (*p*=0.440 and *p*=0.742, respectively) (Table 3).

### 3.3. Factors Influencing the CH

Multivariate linear regression analysis showed that CH was positively associated with CCT (*p* < 0.001) and negatively associated with IOPcc (*p* < 0.001). In a “fixed model,” the CH was found to significantly increase on average 0.02 mmHg for each increase of one micron of CCT, whereas it significantly decreased on average 0.21 mmHg for each increase of 1 mmHg of IOPcc (Table 4).

### 3.4. Factors Influencing the CRF

In multivariate linear regression analysis, CRF was positively associated with CCT (*p* < 0.001) and IOPcc (*p*=0.014). In a “fixed model,” the CH was found to significantly increase on average 0.02 mmHg for each increase of one micron of CCT, whereas it significantly increases on average 0.09 mmHg for each increase of 1 mmHg of IOPcc (Table 4).

## 4. Discussion

The authors present a cross-sectional study where they explored the corneal structural and biomechanical differences between subjects with and without type 2 DM. Our results revealed that IOPcc and CCT were the main parameters associated with corneal biomechanical properties, whereas DM type 2 was not a significant influencing factor. All results were confirmed on our multivariate assessments, adjusting for relevant confounders.

Since the publication of the first study, by Goldich et al. [11] in 2009, several other studies have addressed the effect of hyperglycemia on the corneal biomechanical properties of diabetic patients (Table 5). This subject has special clinical relevance for the growing incidence of diabetes worldwide and also the relevance that the cornea has in the measurement of IOP.

Previous work of Sady and colleagues [9] showed that hyperglycemia causes an increase in advanced Maillard products and oxidative stress resulting in increased collagen crosslinking. Moreover, they found that there was a decrease in the solubility of collagen by pepsin which could explain a reduced turnover of collagen and a consequent increase in corneal thickness and stiffness. However, it is important to note that CH or CRF does not reflect stiffness of corneal tissue [5]. With aging, there is also an accumulation of glycation end products and crosslinking of collagen molecules with increasing stiffness [28]; however, the reduction in the amount of proteoglycans and glycosaminoglycans of the extracellular matrix leads to a reduction in viscoelasticity and CH [18, 29]. On the other hand, in patients with diabetes, the washout of proteoglycans and glycosaminoglycans is reduced because they are more strongly connected and this is believed to increase the viscoelasticity and CH [14].

As pointed out previously in the introduction, the results regarding the influence of diabetes mellitus on the corneal biomechanics have not been consistent throughout the studies. One of the main reasons for this might be the high heterogeneity in subject characteristics across studies, in particular, type of DM and severity of DR. For example, Kotecha et al. [10] divided adult diabetic patients according to the type of DM, while other studies did not specify [11] or mix [12, 14, 16] type 1 and 2 patients without accounting for the important differences between them, such as DM duration. In the study by Kotecha and colleagues, the type 1 DM group was found to have significantly greater CH and CRF when compared to DM type 2 and non-DM subjects, with no statistical difference between the last two groups [10]. It is noteworthy that type 1 DM adult subjects had longer duration of DM in comparison with type 2 DM patients. In turn, two studies [26, 27] investigating corneal biomechanics in children with type 1 DM of short-term duration (<10 years) did not find any difference compared to controls (Table 5). In our study, only type 2 DM patients were included but the differences from controls did not reach statistical significance, as in Kotecha et al.'s study.

In a recent population-based epidemiologic study, Schweitzer et al. [18] found that, in analyses adjusted for age, sex, and IOP, DM was associated with higher CH and CRF values; however, the effect was no longer significant after multivariate adjustment. According to the authors, these findings could be explained by a relatively small sample size of diabetic patients or a confounding effect of plasma LDL cholesterol. In our study, there were also no statistically significant differences between groups which might also reflect the relatively small size of the sample.

The large numbers of studies addressing corneal biomechanical behavior have faced its complexity and highlighted the importance of IOP as a major confounding variable in the assessment of corneal biomechanics using an air-puff stimulus [5]. In fact, our multivariate regression analysis confirmed the IOPcc and the CCT as the main parameters associated with corneal biomechanics properties. This is in line with previous studies that found CH to be positively associated with CCT and negatively associated with IOPcc, whereas CRF positively correlated with CCT and IOPcc [1, 21]. It is also important to recognize that the diabetes itself may also affect IOP and CCT [30]; therefore, we believe that including these covariates in the statistical models increased the confidence of our results and provided more robust conclusions. Importantly, none of the previous works who reported lower CH in diabetics adjusted CH or CRF for IOP, CCT, or age [16, 17, 19].

Scheler et al. [14] was the first to report that patients with poor glycemic control (HbA1c > 7%) had greater values of CH and CRF, controlling for IOP and CCT, compared to controlled DM (HbA1c <7%) and non-DM patients. Similarly, Yazgan et al. [15] reported the same results. Unfortunately, none of the studies provided information on disease duration of each DM group. In our study, longer DM duration was associated with greater HbA1c levels and presence of retinopathy, as expected; nevertheless, the sample included a low number of patients with prolonged DM duration (e.g., > 20 years) and advanced DR which might have influenced the results.

Our analysis failed to demonstrate a significant relationship between CH or CRF and age [29] as described in the literature; however, the CH and CRF values were smaller than other populations with younger samples (Table 5). The lack of a correlation may stem from the cross-sectional nature of our study and, also, the elderly population with short age-range included.

Finally, as DM diagnosis, especially type 2, depends on various factors such as knowledge of risk factors and access to the health system, the real time from onset to diagnosis might be unknown in some patients. This is particularly relevant, as corneal changes might correlate with duration of DM and glycemic control. In our study, all patients regularly attended primary care physicians which might have reduced the selection bias.

In conclusion, the CCT and IOPcc were found to be the main variables that affect corneal biomechanical properties both in diabetic and controls, whereas type 2 DM had no significant effect. The ORA has proven to be an easy-to-use tool that can be incorporated into daily clinical practice to provide important data in patient assessment. Further prospective studies with larger samples and control of confounding factors are required to better understand the relationship between long-term poor glycemic control and corneal biomechanics changes.

## Figures and Tables

**Table 1:** Demographic and clinical characteristics of the study population.

	DM group (*n*=60)	Non-DM group (*n*=48)	*p*
Age (y)	72.38 ± 5.66	70.21 ± 6.45	0.065^1^
Female (*n*)	38 (63.3%)	30 (62.5%)	0.929^3^
Right eyes (*n*)	30 (50.0%)	19 (39.6%)	0.280^3^
BMI (kg/m^2^)	27.91 ± 4.01	27.97 ± 5.01	0.943^1^
Smoking history (*n*)	14 (23.3%)	18 (37.5%)	0.109^3^
HbA1c levels (%)	7.02 ± 1.13	5.54 ± 0.35	<0.001^*∗*^^2^
Duration of diabetes (y)	10.98 ± 8.03	n/a	n/a
DR stage (*n*)			
NPDR absent	42 (70.0%)		
NPDR mild-moderate	10 (16.7%)	n/a	n/a
NPDR severe-PDR	8 (13.3%)		
Oral antidiabetic agents (*n*)	56 (93%)	n/a	n/a
Insulin treatment (*n*)	15 (25%)	n/a	n/a

Data were derived from ^1^independent samples *t*-test, ^2^Mann–Whitney *U*-test, and ^3^chi-squared test. Continuous variables are reported as mean ± standard deviation. ^*∗*^*p* < 0.05 represents statistical significance. DR, diabetic retinopathy; NPDR, nonproliferative DR; PDR, proliferative DR; n/a, not applicable; y, years.

**Table 2:** Ocular characteristics and ORA measurements of the study population.

	DM group (*n*=60)	Non-DM group (*n*=48)	*p*
Axial length (mm)	22.98 ± 0.94	22.91 ± 0.75	0.683^1^
Km (D)	44.11 ± 1.54	44.34 ± 1.57	0.434^1^
Corneal astigmatism (D)	1.01 ± 0.75	0.78 ± 0.49	0.192^2^
CCT^a^ (*μ*m)	557.75 ± 34.72	558.08 ± 30.10	0.958^1^
IOP-GAT (mmHg)	17.73 ± 2.86	16.77 ± 2.66	0.076^1^
IOPg (mmHg)	15.60 ± 3.19	15.24 ± 3.30	0.576^1^
IOPcc (mmHg)	16.28 ± 2.29	16.07 ± 3.29	0.733^1^
CH (mmHg)	10.20 ± 1.45	10.08 ± 1.22	0.637^1^
CRF (mmHg)	10.26 ± 1.49	10.05 ± 1.32	0.439^1^
WS	8.11 ± 1.21	8.30 ± 1.08	0.368^2^

Data were derived from ^1^independent samples *t*-test, ^2^Mann–Whitney *U*-test, and ^3^chi-squared test. Continuous variables are reported as mean ± standard deviation. ^*∗*^*p* < 0.05 represents statistical significance.^a^CCT measured by using Pentacam at corneal vertex. CCT, central corneal thickness; CH, corneal hysteresis; CRF, corneal resistance factor; GAT, Goldmann applanation tonometry; IOP, intraocular pressure; Km, mean keratometry; mm, millimeters; n/a, not applicable; WS, waveform score; y, years; *μ*m, micrometer.

**Table 3:** Subgroup analysis of diabetic patients.

		Age (y)	Female (*n*)	DM duration (y)	HbA1c (%)	DR presence (*n*)	CCT^a^ (*μ*m)	IOPcc (mmHg)	CH (mmHg)	CRF (mmHg)
Duration of diabetes (y)	<10 (*n*=30)	73.30 ± 5.51	18 (60%)	4.90 ± 2.37	6.64 ± 0.75	4 (13%)	556.73 ± 38.15	16.41 ± 3.23	9.92 ± 1.29	9.96 ± 1.54
	≥10 (*n*=30)	71.47 ± 5.75	20 (67%)	17.07 ± 7.00^*∗*^	7.41 ± 1.32^*∗*^	14 (47%)	558.77 ± 31.54	16.16 ± 3.39	10.49 ± 1.57	10.57 ± 1.38

HbA1c levels (%)	<7.0 (*n*=31)	72.90 ± 5.21	18 (58%)	7.58 ± 5.75	6.20 ± 0.47	5 (16%)	553.74 ± 39.15	16.16 ± 3.50	10.02 ± 1.57	9.99 ± 1.49
	≥7.0 (*n*=29)	71.83 ± 6.15	20 (69%)	14.62 ± 8.60^*∗*^	7.91 ± 0.95^*∗*^	13 (45%)	562.03 ± 29.33	16.42 ± 3.10	10.40 ± 1.32	10.56 ± 1.46

DR stage	No (*n*=42)	72.95 ± 5.66	27 (64%)	8.38 ± 6.09	6.85 ± 1.05	—	555.69 ± 36.16	16.76 ± 3.35	10.07 ± 1.48	10.26 ± 1.62
	Yes (*n*=18)	71.06 ± 5.58	11 (61%)	17.06 ± 8.87^*∗*^	7.42 ± 1.26^*∗*^		562.56 ± 31.53	15.17 ± 2.92	10.52 ± 1.37	10.27 ± 1.15

Continuous variables are reported as mean ± standard deviation. ^*∗*^*p* < 0.05 represents statistical significance. ^a^CCT measured by using Pentacam at corneal vertex. CCT, central corneal thickness; K, keratometry; mm, millimeters; n/a, not applicable; WS, waveform score; y, years; *μ*m, micrometer.

**Table 4:** Multivariate regression analysis of the relative effects of clinical and ocular characteristics on corneal biomechanical parameters: corneal hysteresis (CH) and corneal resistance factor (CRF).

Parameter	CH		CRF	
	B (95% CI)	*p*	B (95% CI)	*p*
Age (y)	−0.001 (−0.04 to +0.03)	0.965	−0.003 (−0.04 to +0.04)	0.879
Gender (male)	−0.24 (−0.64 to +0.17)	0.250	−0.303 (−0.80 to +0.19)	0.231
CCT (*μ*m)	+0.02 (+0.01 to +0.02)	<0.001^*∗*^	+0.02 (+0.01 to +0.03)	<0.001^*∗*^
IOPcc (mmHg)	−0.21 (−0.27 to −0.15)	<0.001^*∗*^	+0.09 (+0.02 to +0.16)	0.014^*∗*^
DM duration				
Non-DM	—	—	—	—
DM2 < 10 y	−0.06 (−0.53 to +0.41)	0.792	−0.08 (−0.66 to +0.50)	0.792
DM2 ≥ 10 y	+0.411 (−0.05 to +0.87)	0.080	+0.48 (−0.08 to +1.05)	0.093

Data were derived from generalized linear models. ^*∗*^*p* < 0.05 represents statistical significance. CH, corneal hysteresis; CI, confidence interval; CRF, corneal resistance factor; D, diopters; DM, diabetes mellitus; mm, millimeters; y, years; *μ*m, micrometer. The remaining variables (HbA1c, smoking history, BMI, AL, Km, and IOP-GAT) did not influence the model and were excluded.

**Table 5:** Review of literature comparing corneal biomechanics obtained by ORA in patients with and without diabetes mellitus.

Study (year)	Nr of patients (eyes)		Glaucoma	Age (y)	Female (*n*)	DM diagn. duration	DR stage	HbA1c (%)	CCT (*μ*m), US	IOPcc (mmHg)		CH (mmHg)	CRF (mmHg)
Goldich et al. [11] (2008)	Cont.	40 (40)	No	64 ± 9	19 (48%)	Interview	n.r.	n.r.	530.3 ± 35.9		17.7 ± 4.9	9.3 ± 1.4	9.6 ± 1.6
	DM	40 (40)		61 ± 12	17 (43%)	Duration n.r.			548.7 ± 33.0^*∗*^		16.6 ± 4.4	10.7 ± 1.6^*∗*^	10.9 ± 1.7^*∗*^

Hager et al. [12]^a^ (2009)	Cont.	195 (385)	26%	65 ± 16	206 (58%)^f^	Interview	Most patientsDR	n.r.	542.0 ± 40.0^d^		n.r.	10.4 ± 1.9	n.r.
	DM	50 (99)	20%	70 ± 11	42 (42%)^f^	13 ± 9 y			554.0 ± 50.0^d^			10.7 ± 1.7^*∗*^	

Sahin et al. [16]^b^ (2009)	Cont.	61 (120)	No	53 ± 10	33 (54%)	Interview	n.r.	n.r.	535.5 ± 39.2		15.8 ± 3.2	10.4 ± 1.7	10.4 ± 2.0
	DM	43 (81)		55 ± 12	26 (60%)	14 ± 6 y		7.3 ± 1.5	550.1 ± 40.8^*∗*^		18.8 ± 4.7^*∗*^	9.5 ± 1.8^*∗*^	10.3 ± 1.8

Kotecha et al. [10] (2010)	Cont.	123 (123)	No	54 ± 16	n.r.	Interview	n.r.	n.r.	550.1 ± 32.8		15.9 ± 2.7^e^	10.8 ± 1.7	10.6 ± 1.6
	DM 1	13 (13)		42 ± 11	18 (30%)	24 ± 15 y		7.3 ± 0.6	551.1 ± 27.2		15.4 ± 2.5^e^	12.4 ± 1.7^*∗*^	12.5 ± 2.0^*∗*^
	DM 2	48 (48)		62 ± 11		12 ± 10 y		7.2 ± 1.4	550.0 ± 40.9		16.2 ± 2.5^e^	10.9 ± 1.9	11.5 ± 2.1

Castro et al. [13] (2010)	Cont.	25 (40)	Yes	66 ± 15	16 (64%)	Interview	No DR	n.r.	546.6 ± 37.3		n.r.	7.8 ± 1.7	n.r.
	DM 2	19 (34)		67 ± 9	14 (74%)	Duration n.r.			531.7 ± 31.3			9.1 ± 1.9^*∗*^	

Scheler et al. [14]^c^ (2012)	Cont.	35 (35)	No	61 ± n.r	24 (69%)	Guidelines	n.r.	5.4 ± 0.5	n.r.		n.r.	10.7 ± 1.8	10.6 ± 2.2 n.r.
	DM < 7%	14 (14)		66 ± n.r	14 (45%)	Duration n.r		6.0 ± 0.8				n.r.	12.2 ± 2.1^*∗*^
	DM ≥ 7%	17 (17)						8.6 ± 2.4				11.2 ± 2.1	

Celik et al. [15] (2014)	Cont.	74 (74)	No	58 ± 10	50 (68%)	Guidelines	All DR stages	5.2 ± 0.6	551.4 ± 3.1		16.6 ± 0.4	9.0 ± 0.2^*∗*^	9.0 ± 0.2^*∗*^
	DM 2 < 7%	74 (74)		58 ± 10	40 (54%)	Duration n.r.		6.3 ± 0.3	543.0 ± 3.2		17.1 ± 0.4	9.8 ± 0.2^*∗*^	10.1 ± 0.2^*∗*^
	DM 2 ≥ 7%	82 (82)		58 ± 9	56 (68%)			9.9 ± 1.5	566.4 ± 3.0^*∗*^		18.2 ± 0.3^*∗*^	10.9 ± 0.2^*∗*^	11.9 ± 0.2^*∗*^

Pérez-Rico et al. [17] (2015)	Cont.	41 (41)	No	61 ± 9	31 (76%)	Guidelines	n.r.	5.2 ± 0.6	516.1 ± 34.0		14.6 ± 3.7	11.4 ± 1.7	10.5 ± 1.8
	DM 2 < 7%	40 (40)		62 ± 10	23 (58%)	12 ± 9 y		6.3 ± 0.3	561.3 ± 34.7		14.7 ± 2.7	10.9 ± 1.4	11.2 ± 2.0
	DM 2 ≥ 7%	54 (54)		60 ± 13	32 (59%)	15 ± 11 y		9.9 ± 1.5	565.2 ± 38.6		18.4 ± 3.8^*∗*^	10.2 ± 1.8^*∗*^	11.1 ± 2.0

Schweitzer et al. [18] (2016)	Cont.	695 (695)	No	>74^g,h^	-^g^	Guidelines	n.r.	n.r.	548.9 ± n.r.		-^g^	9.3 ± *n*.r.	9.6 ± *n*.r.
	DM 2	137 (137)				Duration n.r			558.2 ± n.r.			9.8 ± *n*.r.^*∗*^	10.4 ± *n*.r.^*∗*^

Bekmez and Kocaturk [19] (2018)	Cont.	50 (50)	No	62 ± 12	26 (52%)	—	—	—	—		16.0 ± 3.1	10.5 ± 1.7	10.5 ± 1.7
	DM 2	50 (50)		63 ± 9	25 (50%)						17.8 ± 3.6^*∗*^	9.9 ± 1.5	10.4 ± 1.6

Kara et al. [26] (2013)	Cont.	50 (50)	No	15 ± 2	31 (62%)	Guidelines	No DR	n.r.	559.0 ± 22.0		15.1 ± 2.7	12.5 ± 1.5	11.9 ± 1.5
	DM 1	46 (46)		14 ± 2	26 (57%)	6 ± 3 y		10.4 ± 2.4	555.0 ± 26.0		15.5 ± 3.4	12.3 ± 1.3	12.4 ± 1.7

Nalcacioglu-Yuksekkaya et al. [27] (2014)	Cont.	74 (74)	No	13 ± 3	48 (65%)	Guidelines	No DR	n.r.	n.r.		15.3 ± 3.4	10.7 ± 1.7	10.5 ± 1.6
	DM 1	68 (68)		13 ± 3	34 (50%)	5 ± 3 y		8.3 ± 2.0			15.8 ± 3.0	10.8 ± 1.5	10.9 ± 1.9

^*∗*^Statistical significant difference at *p* < 0.05. CCT, central corneal thickness; diagn., diagnosis; DR, diabetic retinopathy; SD, standard deviation; US, ultrasound paquimetry; y, years; mmHg, millimeters of mercury. ^a^Retrospective study. ^b^The study included 22 type 1 DM and 21 type 2 DM patients. ^c^The study included 3 type 1 DM and 28 type 2 DM patients. ^d^CCT was measured with Orbscan; ^e^IOPcc was not reported, and the values of IOP were measured with a dynamic contour tonometer. ^f^Number of eyes included in the study. ^g^CCT, CH, and CRF values were adjusted for age, sex, and IOP; ^h^Mean age was comparable between groups; ^i^Values are reported as mean ± standard error.

## Data Availability

The data used to support the findings of this study are available from the corresponding author upon request.
